# MRI for restaging pancreatic cancer

**DOI:** 10.1186/s13244-026-02422-1

**Published:** 2026-10-13

**Authors:** Giulia A. Zamboni, Federica Omboni, Irene Panozzo

**Affiliations:** Department of Diagnostics and Public Health, Institute of Radiology, University of Verona, Policlinico GB Rossi, Verona, Italy

**Keywords:** Pancreatic neoplasms, Adenocarcinoma, Neoadjuvant therapy, Magnetic resonance imaging

## Abstract

**Abstract:**

Pancreatic adenocarcinoma is a tumor with a dismal prognosis, as the majority of patients are diagnosed when the tumor is not resectable. Neoadjuvant treatment has significantly improved prognosis, but assessing treatment response can be challenging for radiologists. Given the fibrotic nature of the primary tumor, differentiating between residual viable tumor and fibrotic response can be difficult. MRI can have a role in reassessing the primary tumor and disease spread. In this review, we discuss MR techniques that can be used for this assessment, including routinely used sequences (e.g., DWI) and sequences that are not yet routinely included in pancreatic protocols (e.g., T1 mapping, ECV imaging, and USPIO contrast for nodal staging).

**Key Points:**

***Question*** Imaging assessment of the response of pancreatic adenocarcinoma to neoadjuvant treatment is difficult and still has limited accuracy.

***Findings*** Different MRI sequences and techniques have been suggested as possibly improving this assessment, reflecting on better patient management.

***Critical relevance statement*** We summarize the available evidence on MRI for restaging pancreatic carcinoma after neoadjuvant treatment.

**Graphical Abstract:**

**Figure:**
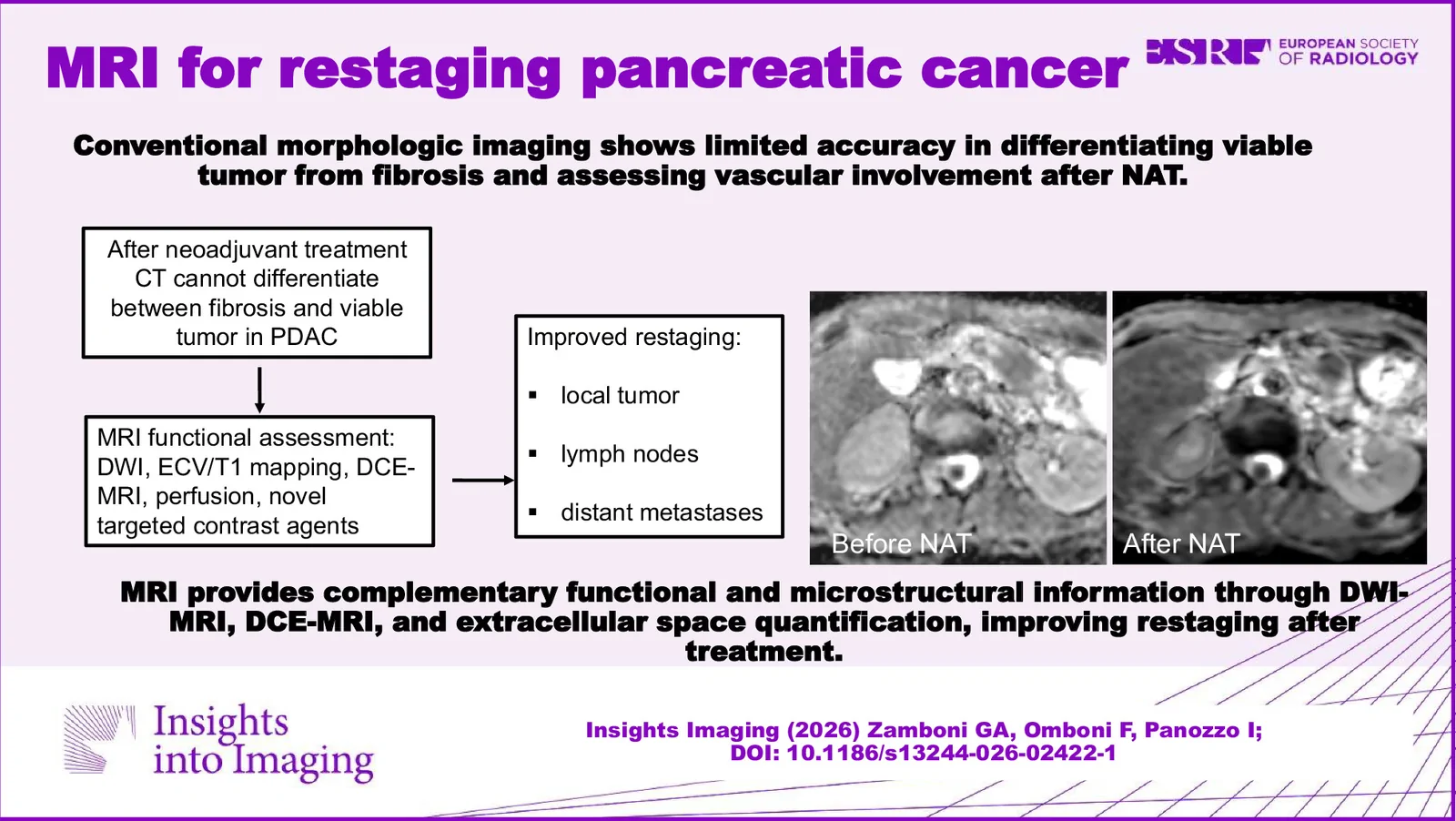

## Introduction

Assessing the response after neoadjuvant therapy (NAT) remains an important challenge in modern imaging. Considering that pancreatic adenocarcinoma is an aggressive cancer with an estimated 5% 5-year survival [1] and that only 15–20% of pancreatic adenocarcinomas are diagnosed when upfront resectable, correct categorization of patients who respond to neoadjuvant treatment becomes fundamental, granting the possibility to undergo radical surgery with higher survival chances [2].

While the assessment of distant metastatic spread is considered feasible, particularly with dedicated MRI for liver metastases detection, local staging remains challenging, with imaging frequently overestimating tumor burden even in pathology-proven R0 resections [3]. Consequently, surgical exploration is the preferred approach to assess tumor resectability, recommended in patients with radiologically stable disease and permissive CA19-9 levels (e.g., below 500 IU/mL) [4–7]; however, it may undeniably lead to inappropriate resections in patients with vascular invasion and advanced disease, or exclude potentially resectable patients from achieving an R0 resection [2].

One of the most important issues in the assessment of response is the challenge posed by estimating a fibrotic response in a lesion characterized by pre-existing fibrosis [8]: in this context, where the pancreatic lesion can undergo little or no size reduction even if tumoral cells actually respond, relying solely on a morphological evaluation inevitably leads to inaccurate staging of residual disease [3].

This is particularly evident in CT restaging after NAT, where, despite the high spatial resolution, low accuracy in treatment response assessment persists [9, 10].

To overcome this bias, the focus has begun to shift to functional imaging. The role of PET-CT has been evaluated [11, 12], with NCCN guidelines recommending that metabolic imaging (PET-CT) be considered before and after neoadjuvant treatment to assess metabolic response and restaging. However, this modality is characterized by low accessibility and low spatial resolution, which could hinder local staging [2, 11, 12].

In this context, MR could be an important asset in evaluating the response to NAT. While not inferior to CT in assessing lesion size and vascular invasion [13, 14], it could provide additional information on the microenvironment and other functional parameters. In particular, DWI imaging has been considered promising for assessing response in pancreatic adenocarcinoma: the ability to evaluate both the extracellular compartment and residual areas of tumoral hypercellularity, thereby differentiating them from the concomitant fibrotic response, lays the foundation for outperforming a pure morphological evaluation [2, 11, 12].

Also, further MR imaging applications focused on microarchitectural evaluation can open new possibilities for evaluating response after neoadjuvant therapy in pancreatic adenocarcinoma.

## Pathology after NAT

The main challenges faced by radiologists when assessing pancreatic cancer response after NAT lie in the pathologic features of pancreatic adenocarcinoma before and after therapy.

Naive pancreatic adenocarcinoma is usually composed of sparse cancerous cells surrounded by a dense fibrotic desmoplastic stroma [8, 15], which demonstrates the typical features of fibrotic tissue at traditional imaging; tumor response to NAT consists mainly of a fibrotic response [15], which at imaging will appear somewhat indistinguishable from the pre-existing fibrosis. Adding further complexity, cancerous cells do not follow the classic pattern of peripheral shrinkage but usually regress heterogeneously throughout the mass: tumors do not undergo a classical size reduction, and areas with different cancer burdens can coexist within the same mass [8]. That, together with the atrophy and fibrous reaction near tumor margins [8, 15], makes it very difficult to correctly assess tumor shrinkage and differentiate viable tumor tissue. Moreover, radiation therapy induces tissue inflammation and peripancreatic fat edema that might further confuse the imaging pattern [2].

## Imaging challenges after NAT

**Fig. 1:**
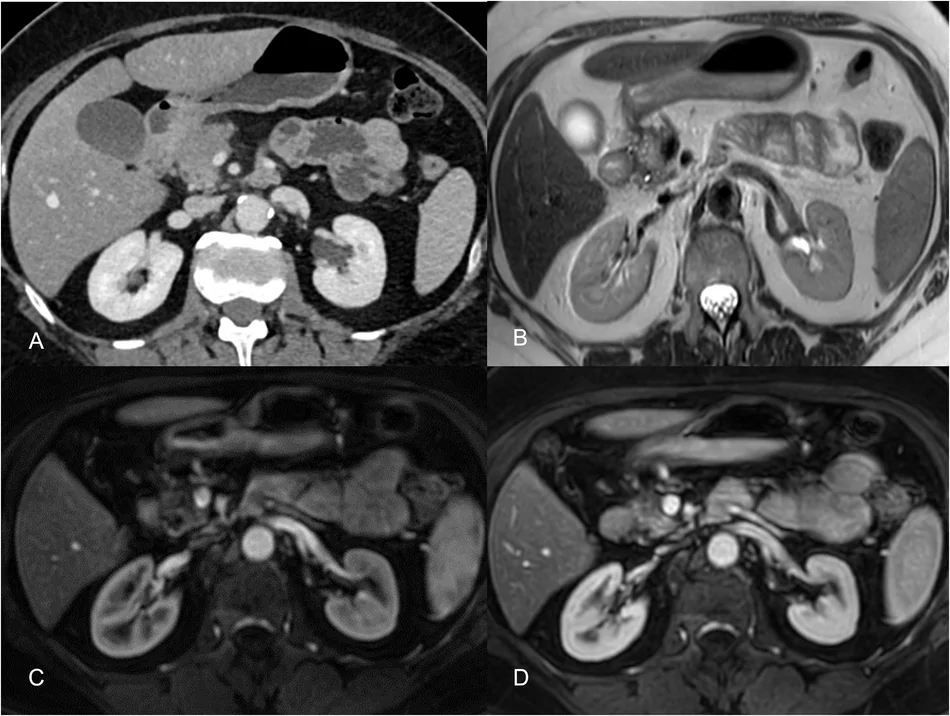
Axial contrast-enhanced CT (**A**) shows a hypodense lesion in the pancreatic head encasing the superior mesenteric vein, causing its deformation and narrowing. Stage according to NCCN: borderline resectable. Post-NAT MRI T2 (**B**), arterial phase (**C**), and portal-venous phase (**D**) images show that the lesion is reduced in size with re-expansion of the superior mesenteric vein and residual contact between the mass and the SMV < 180°. The partial response to treatment downstaged the lesion as resectable according to NCCN. The patient underwent pancreaticoduodenectomy; pathology of the resected specimen confirmed residual infiltration of the SMV and negative margins (R0)

Histological features account for the most important challenges when assessing NAT response on imaging: subtle changes in fibrotic tissue are difficult to distinguish, and conventional imaging will demonstrate fibrotic tissue as hypovascular after contrast administration, with progressive enhancement in the late post-contrast phase, making viable tumor and fibrotic response indistinguishable (Fig. 1).

In most cases, the tumor will not shrink after treatment, remaining stable compared to baseline imaging, making dimensional criteria, e.g., RECIST 1.1, unreliable for defining a potential response [2, 3, 12]. In this context, lack of size reduction does not equal absence of response/residual viable tumor and could coexist with a tumor bed completely replaced by fibrosis.

The issue persists in the estimation of vascular invasion, making it difficult, or even impossible, in most cases: the contact with arteries/veins could be unmodified, with unchanged vessel distortion, and this feature could coexist with the absence of actual vascular invasion and only residual fibrosis surrounding vascular structures [9, 10, 12].

In a clinical scenario where morphological assessment of size criteria, such as RECIST 1.1, and evaluation of vascular involvement become unreliable, being unable to guarantee an accurate estimate of response to therapy, non-morphological imaging techniques become an important alternative for restaging [11].

**Fig. 2:**
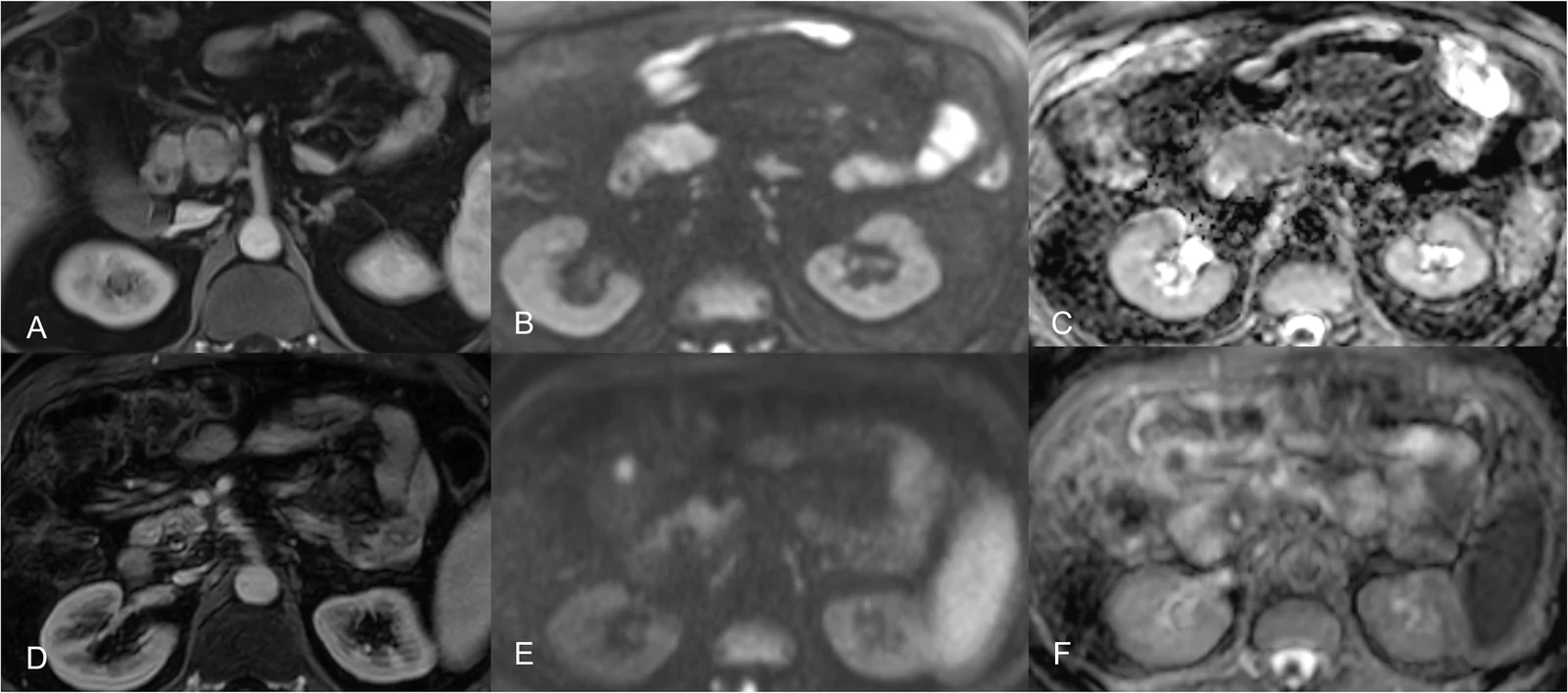
Baseline MRI arterial phase (**A**) shows a solid lesion in the pancreatic head with restricted diffusion (**B**, **C**). After NAT, the lesion is reduced in size (**D**), with a smaller area of restricted diffusion (**E**, **F**). The patient underwent resection, and pathology showed negative margins (R0)

**Fig. 3:**
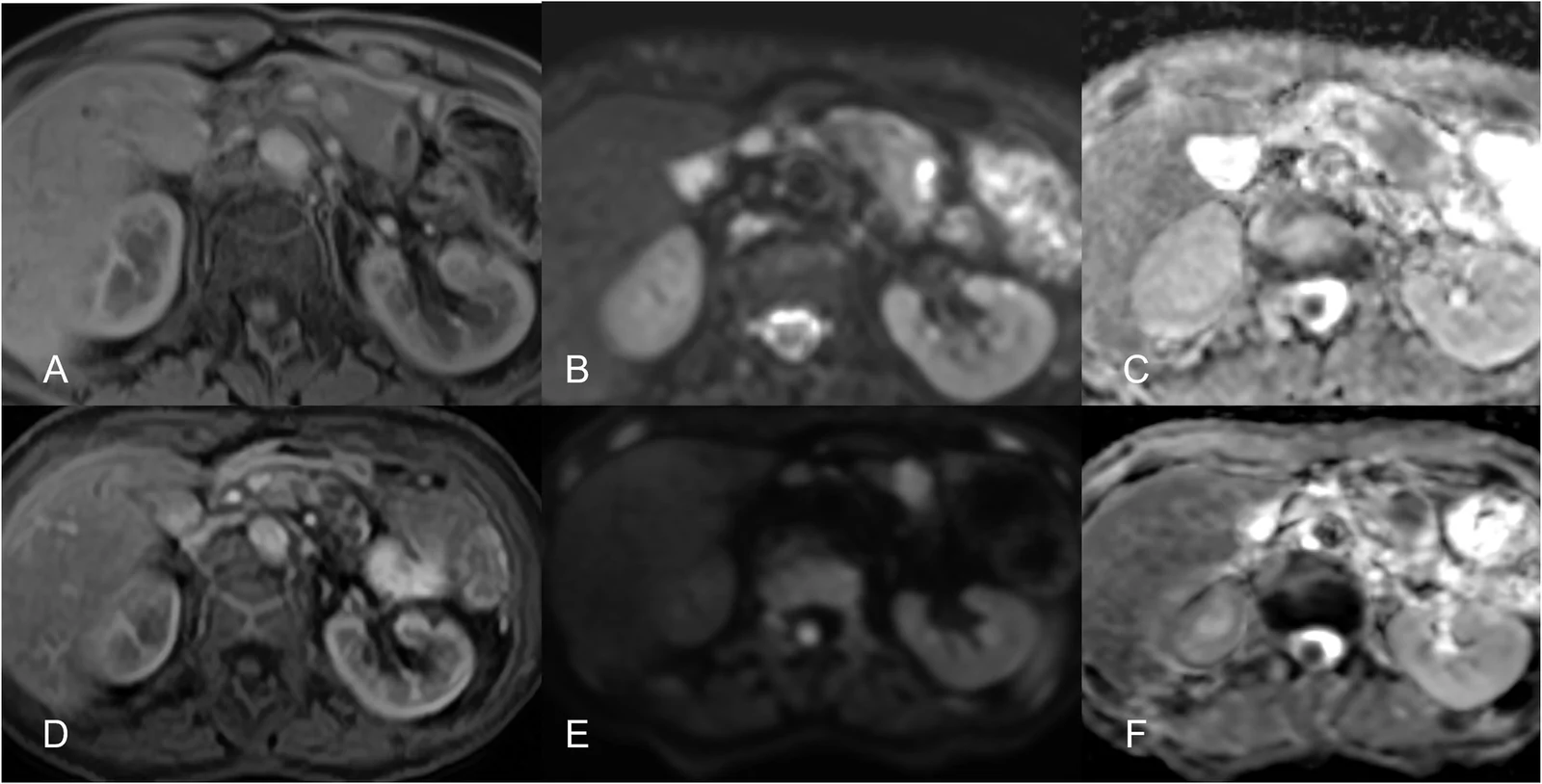
Baseline MRI arterial phase (**A**) shows a large solid hypovascular lesion in the body-tail with restricted diffusion (**B**, **C**). After NAT, the lesion is markedly reduced in size (**D**), with a visible fat plane around the vessels and a smaller area of restricted diffusion (**E**, **F**). The patient, however, did not undergo resection because of poor clinical conditions and showed progression at subsequent follow-up imaging

**Fig. 4:**
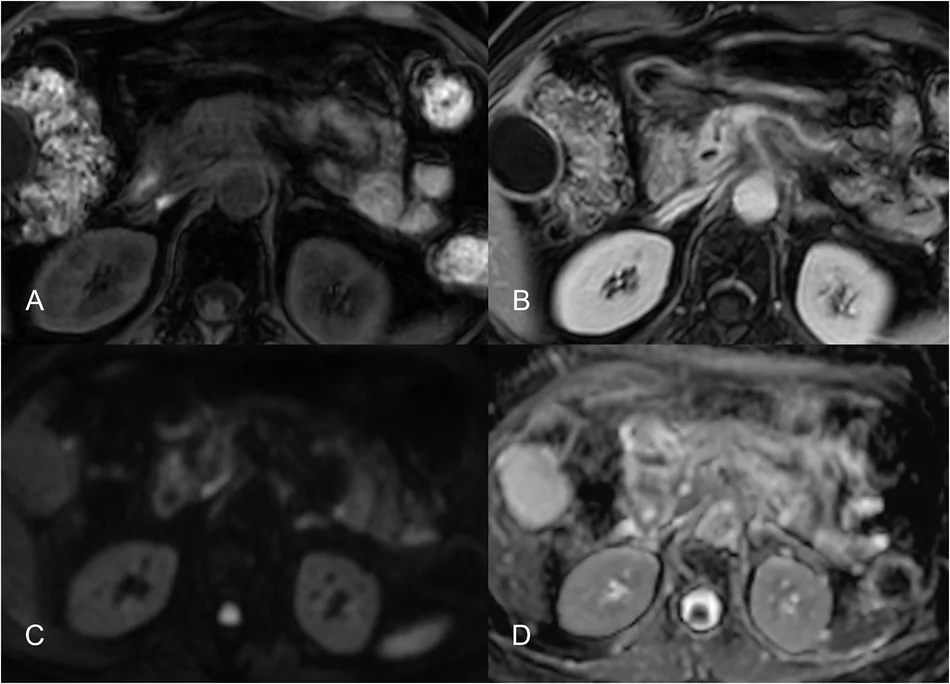
Post-NAT MRI non-contrast T1 (**A**) and arterial phase (**B**) images show solid hypovascular tissue encasing the celiac axis, with no significant restricted diffusion (**C**, **D**). The patient underwent pancreaticoduodenectomy; pathology of the resected specimen showed extensive fibrosis with sparse and very few carcinomatous glands, with R0 margins

**Fig. 5:**
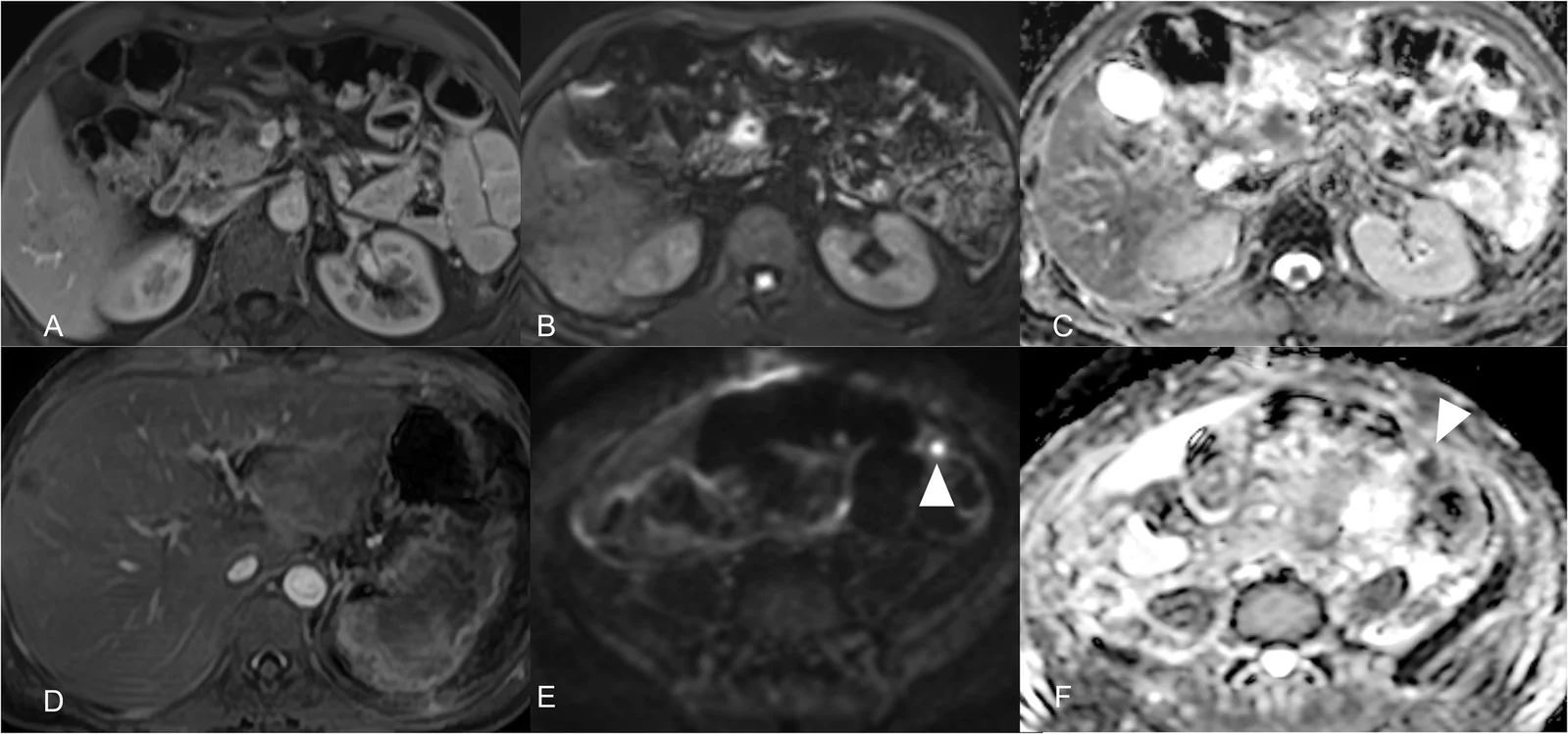
Baseline MRI arterial phase (**A**) shows a solid lesion with restricted diffusion (**B**, **C**) encasing the SMV. During NAT, the patient developed liver metastases (**D**) and ascites with solid peritoneal nodules compatible with carcinomatosis (arrowhead in **E**, **F**)

In this regard, MR offers opportunities to investigate tumor microarchitecture that largely exceed those of CT, potentially overcoming reported standard morphological limitations in response assessment. Through DWI imaging, extracellular space assessment techniques, and novel targeted contrast media, MRI could enable the detection of subtle changes in tumor microarchitecture and microenvironment, potentially improving the accuracy of post-treatment response evaluation (Figs. 2–5).

## Indications and techniques

According to the NCCN Guidelines [16], restaging of pancreatic adenocarcinoma can be performed either with CT or MR with a dedicated pancreatic protocol; while CT remains the currently preferred method, MR is considered as an alternative for the study of indeterminate liver lesions, adenocarcinomas not visible on CT (such as isodense PDAC), or in case of severe allergy to iodinated contrast.

Some authors recommend MRI as a mandatory step for liver metastases evaluation, both in borderline and locally advanced PDAC, before and after neoadjuvant treatment [2].

Traditional MR pancreatic dedicated protocols include T2-weighted sequences with and without fat suppression, T1- in and out of phase, DWI with high b-values, T1-weighted fat-suppressed gradient echo before and after IV gadolinium administration, and MRCP (Table 1).

**Table 1:** MRI protocol

Traditional MR protocol			
**Sequence**	**Plane**	**Section thickness**	
T2-weighted single-shot fast spin echo (SSFSE)	Coronal ± axial	< 6 mm	
T1-weighted in-phase and opposed-phase gradient echo (GRE)	Axial	< 6 mm	
T2-weighted fat-suppressed fast spin echo (FSE)	Axial	< 6 mm	
Diffusion-weighted imaging large FOV (DWI)	Axial	< 6 mm	
Pre and dynamic post IV contrast injection T1-weighted fat-suppressed gradient echo GRE (pancreatic, portal venous, equilibrium phase)	Axial	Thinnest possible 2–3 mm	
T2-weighted MR cholangiopancreatography (MRCP) preferably 3D, fast relaxation spin echo sequence FRFSE	Coronal	< 3 mm	
Table adapted from NCCN guidelines [12].			
**Other techniques**			
**Sequence**	**Plane**	**Thickness**	**Purpose**
5 min post IV contrast modified Look-Locker inversion recovery sequences using single-shot steady-state free precession readout SSSFP	Axial	10 mm (2 slices)	T1 mapping
Three-dimensional fat-suppressed T1-weighted gradient-echo volumetric interpolated breath-hold examination with motion-resolved compressed-sensing reconstruction (CS-VIBE)	Axial	Thinnest possible 2–3 mm	DCE-MRI
Fat-suppressed T2*-weighted multi gradient echo (mGRE) sequence, 24–36 h after administration of Femuroxtran-10	Coronal ± axial	Thinnest possible 2–3 mm	USPIO–LN staging
Sequences adapted from Fukukura et al [13] and Litjens et al [14]			

## Local restaging

Local disease restaging after neoadjuvant therapy remains a major concern and presents several challenges for radiologists, since the distinction between viable tumor and treatment-induced fibrosis can be difficult. Current clinical approaches continue to rely primarily on morphologic response criteria (e.g., RECIST on standard CT/MRI), biochemical markers such as CA19-9, and metabolic imaging with FDG-PET, but macroscopic changes in mass, size or morphology that occur after successful therapies may not reflect the true proportion of the tumor response, given the characteristic non-expanding growth pattern of PDAC, potentially resulting in a delay in clinical decisions [17]. Conversely, microstructural tumoral changes, such as necrosis, edema, inflammation, and fibrosis, may have a substantial impact on quantitative parameters evaluated through MR imaging, especially Diffusion-weighted MRI (DWI), which is increasingly emerging as a promising tool for restaging pancreatic ductal adenocarcinoma following neoadjuvant chemotherapy [11, 18–20].

Despite their ability to detect microstructural changes, DWI and DWI-derived quantitative parameters are not yet formally integrated into standardized response criteria, as only a few studies have investigated their role in post-NAT PDAC assessment.

In DWI-MRI, the signal intensity reflects the impeded (or restricted) diffusion of water molecules, resulting in a low apparent diffusion coefficient (ADC) on ADC maps and a high signal intensity on DWI images. An increase in ADC values is generally associated with a good response to treatment, while stable or decreasing values may suggest a poor response, requiring to redefine the therapeutic approach.

A 2024 systematic review and meta-analysis by Bilreiro et al, including 7 studies and 161 patients, reported high sensitivity (91.6–100%) and moderate-to-high specificity (62.5–94.7%) for DWI in assessing response to NAT using histopathology or clinical outcomes as reference standards. However, substantial overlap in pre- and post-treatment ADC values between responders and non-responders, combined with study heterogeneity and risk of bias, limits the reliability of these findings [21].

Dalah et al found a significant correlation between the change in ADC values before and after treatment and pathological treatment response in 25 patients with PDAC following neoadjuvant chemoradiation. Analysis of pre- and post-neoadjuvant therapy ADC histograms showed a shift toward higher ADC values in tumors that pathologically showed a good response to treatment [22].

Similarly, Okada et al reported a significant correlation between whole-tumor ADC values (before and after treatment) and histological tumor cell destruction rate after neoadjuvant therapy, suggesting that post-treatment whole-tumor ADC values may be a predictor of R0 resectability in patients with borderline-resectable PDAC, although disclosing limits regarding the small sample size, the single institution-based retrospective nature of the study, and the short follow-up period [23].

In a retrospective study including 25 patients by Qu et al, a significant increase in after-NAT ADC values and intravoxel incoherent motion (IVIM) parameters (true diffusion coefficient D and perfusion fraction f) was observed in the response group compared to the non-response group, associated with a significant improvement in OS and PFS, suggesting that DWI may provide useful information about the efficacy and prognosis of NAT in borderline-resectable patients [18].

Despite these promising results, significant limitations persist, and findings across studies are not consistent, as illustrated by Chen et al, who found that although ADC values increased after NAT, these changes did not reliably predict tumor regression grade or margin status [24].

DWI also has several technical limitations that affect its use in clinical practice [25]. Image quality can be reduced by susceptibility artifacts arising from adjacent air-filled structures, motion artifacts induced by respiratory movements, and magnetic field inhomogeneity. Other issues include distortion, ghosting, and a low signal-to-noise ratio (SNR), which can reduce accuracy and reliability.

Also, the lack of standardized imaging protocols across institutions may hinder the clinical application of DWI in pancreatic cancer, since disparities in b-values, ADC calculations, and the placement of regions of interest (ROI) result in inconsistencies in diagnostic outcomes [25].

By standardizing imaging methodologies, DWI could become a more reliable tool in pancreatic cancer management, improving diagnostic accuracy and enabling more consistent assessment of treatment outcomes.

Beyond estimating tumor response to NAT based on microstructural changes, MRI may also help determine tumor resectability and the American College of Radiology Appropriateness Criteria (2025) suggest it as a possible primary tool for restaging [26].

Morphological MRI staging and evaluation of residual vascular tumor involvement following NAT remain debated, and the primary evidence comes from a prospective pilot study including 19 patients [27]. Stoop et al investigated the role of the MRI-halo sign in assessing resectability in patients with locally advanced pancreatic cancer after induction chemotherapy. This imaging finding is defined as the replacement of solid perivascular tumor tissue by a zone of fatty-like signal intensity around the involved artery or vein on contrast-enhanced MRI, suggesting treatment-related tumor regression at the vessel interface. The study showed that the MRI-halo sign was not associated with overall resection rate and, among patients who underwent resection, those with an MRI-halo sign did not have a significantly higher R0 resection rate. However, the MRI-halo sign was detected on CE-MRI in 52.6% of patients (10/19), compared to only 10.5% (2/19) on CT (*p* = 0.016), suggesting an additional value of MRI over CT in visualizing perivascular treatment response. Since further validation is needed, current literature data about morphological restaging of PDAC based on MRI findings is not yet part of standard clinical practice.

## Extracellular space assessment techniques

The extracellular space and tumor microenvironment are important in pancreatic adenocarcinoma and become relevant when considering that NAT therapy can induce significant changes in these components, which could be used to assess treatment response.

These techniques could have an important role in predicting tumor response, overcoming purely morphological assessments such as size criteria.

It must be noted that different neoadjuvant treatments act differently and induce different responses on pancreatic adenocarcinoma and on its extracellular space, potentially shifting the composition and ratio of the latter: while FOLFIRINOX response mainly consists of fibrotic tissue deposition in the tumor bed [28], gemcitabine is thought to affect both tumoral cells and microenvironment, and in particular tumor vascularization [29].

In this regard, evaluation of the extracellular space shows promise for tumor response assessment and can be investigated not only through ADC maps but also with other specific MR techniques.

Assessment of extracellular volume (ECV) through T1-mapping techniques has shown promising results. ECV estimation is currently applied in other imaging settings, being an established method to estimate the grade of myocardial fibrosis in cardiac imaging [30]; this application could be translated to pancreatic carcinoma to assess possible modifications of NAT-induced fibrosis in a non-invasive way.

In a previous study by Fukukura et al [31] on 58 patients, ECV fraction was found to be significantly higher in patients who responded to neoadjuvant treatment, with a reported sensitivity of 89.3%, specificity of 90.0%, and accuracy of 89.7%, suggesting it could be a useful tool for estimating tumor response to therapy.

The use of DCE-MRI to assess tumor perfusion and microvascular environment has also been investigated. This technique allows assessment of pharmacokinetic parameters, such as the volume transfer constant (K_trans_), the extravascular extracellular volume fraction (v_e_), and AUC_60_, using dedicated post-processing tools. While showing promising results in studies on patients treated with anti-angiogenetics (e.g., sorafenib) and gemcitabine [29, 32], with higher values of Ktrans and ve in the responders group, this observation was not confirmed in studies of murine models treated with FOLFIRINOX [29]: this technique might therefore be less suitable to address NAT response in regimens that induce less marked effects on tumoral vascularization. Further studies are required to assess the efficacy of different imaging techniques in assessing the response of different NAT regimens. It must also be noted that these imaging techniques require expertise to be applied in clinical practice, potentially limiting their use in non-dedicated centers.

On the other hand, new MR contrast agents, and in particular targeted contrast agents, are currently being investigated at the pre-clinical stage [33–35]. Targeted contrast agents are designed to selectively bind ions, amino acid derivatives, or proteins that are quantitatively altered in the tumor microenvironment. They can act alone or need the adjunct of a secretagogue to bind specific targets.

One example is a type I collagen-targeted MR contrast agent (CM-101), which demonstrates affinity for type I collagen, binding it and being retained by fibrotic tissue; this contrast medium has been tested in murine models, demonstrating better resolution of tumor margins and improving the assessment of FOLFIRINOX response by evaluating the fibrotic tissue deposition in the tumor bed [33]. The role of other new specific targeted contrast agents, such as secretagogue-stimulated zinc secretion MRI [34] and allysine-targeted molecular MRI probe [35], is being tested in mouse models with promising results.

To the best of our knowledge, there are no literature reports on MRI spectroscopy for post-NAT response assessment.

## Nodal restaging

Nodal restaging follows RECIST 1.1 criteria, with complete response defined as a reduction in the short-axis diameter of a pathologic lymph node to less than 10 mm. However, it is known that the rate of nodes below 10 mm positive at pathology can be significant [36–38].

The use of iron-particle-based contrast agents has demonstrated potential for detecting even small metastatic nodes (< 10 mm) that are not considered positive by standard morphological criteria [38, 39]. Thanks to USPIO contrast agents, even small nodes can be detected as metastatic due to the lower accumulation of iron particles in macrophages and consequent lack of hypointensity in magnetic susceptibility sequences such as T2*; reported sensitivities and specificity are respectively 83% and 80–92% from two previous studies by Litjens et al [40] and McDermott et al [41], which revealed positive nodes with mean diameter of 5–5.5 mm. These studies show significantly higher diagnostic performance of USPIO MR in detecting lymph node metastases compared with CT and standard MRI with gadolinium-based contrast agents. Studies on USPIO include small populations, and there is no data on performance in nodal staging after NAT in PDAC: further studies are therefore needed to assess its efficacy after neoadjuvant therapy in pancreatic cancer. Accurate preoperative nodal staging could potentially exclude from resection patients with distant lymph nodal metastases, such as positive para-aortic nodes [38, 42], and aid surgeons in defining the extent of required lymphadenectomy [43].

Regarding DWI, research has primarily focused on primary tumor response, while the role of this technique in assessing regional lymph nodes after NAT remains underexplored. Current available evidence supports DWI in the evaluation of lymph node involvement in treatment-naïve patients: Rong et al [44] investigated the value of intravoxel incoherent motion diffusion-weighted magnetic resonance imaging (IVIM-DWI) for differentiating metastatic from non-metastatic lymph nodes in patients with PDAC. In this prospective study, 15 patients underwent preoperative MRI, followed by surgical resection, with histopathological evaluation of the lymph nodes. Quantitative IVIM parameters, including the apparent diffusion coefficient (ADC), true diffusion coefficient (D), pseudo-diffusion coefficient (D*), and perfusion fraction (f), were compared between metastatic and benign lymph nodes. The authors found that metastatic lymph nodes had significantly lower ADC and D values than non-metastatic nodes, and, among the evaluated parameters, the true diffusion coefficient (D) demonstrated the highest diagnostic performance for identifying nodal metastases, although the findings were limited by the small sample size and the inclusion of only treatment-naïve patients, without evaluation after neoadjuvant therapy.

Additionally, Shi et al [45] developed a multiparametric MRI model to predict small (< 10 mm) lymph node metastases in patients with resected PDAC and suggested that integrating DWI with conventional MRI features may improve the preoperative prediction of small metastatic lymph nodes in PDAC. However, all patients underwent upfront surgery without neoadjuvant therapy, limiting the applicability of these findings to the post-treatment setting.

## Distant staging

Approximately half of the patients with PDAC present with metastatic disease [46–48]: a 2025 Lancet review reported that among metastatic PDAC patients, the liver is involved in 75–80%, peritoneum in 13–30%, lung in 15–18%, and extra-regional lymph nodes in roughly 12% of cases [47].

The detection of liver metastases is crucial after NAT, since the presence or absence of metastatic involvement directly influences surgical eligibility, and resection is generally considered only in patients without metastatic disease.

Although CT remains the primary imaging modality for restaging, MRI provides additional diagnostic value in several clinical scenarios. Several studies reported that MRI offers higher contrast resolution and detects liver metastases more effectively than CT, particularly when assessing subcentimeter lesions in patients with potentially resectable pancreatic ductal carcinoma [46, 49–52].

Yamaguchi et al found that gadoxetic acid-enhanced MRI combined with CT provided higher diagnostic performance than CT alone for detecting liver metastases in patients with potentially resectable PDAC, and additionally suggested the use of an abbreviated MRI protocol, including T2-weighted, DWI, and hepatobiliary phase imaging, with an overall reduction in costs and examination time [46].

In a more recent retrospective study, Jeon et al reported that MRI with DWI achieved higher diagnostic accuracy in characterizing indeterminate or suspicious lesions detected on initial CT in patients with potentially resectable pancreatic ductal carcinoma [48].

Regarding peritoneal metastases, the literature is conflicting: while some studies have reported that MRI is more sensitive than CT for detecting peritoneal disease, others have observed similar performance.

Durán et al reported superior sensitivity of DWI-MRI compared to CT for detecting small peritoneal implants and lesions in anatomically complex regions such as subphrenic spaces, hepatic surface and Morrison’s pouch, lesser sac, small bowel and mesentery, rectouterine pouch and rectovesical pouch, and greater omentum [53]. Conversely, Chia et al reported similar performance for CT and MRI in overall detection and assessment of peritoneal lesions. However, the same group suggested a more thorough evaluation of DWI-MRI for site-specific tumor deposits, particularly those within the mesentery, bowel serosa, perihepatic, and peripancreatic spaces [54]. This is in good agreement with a meta-analysis by van’t Sant et al, which reported that mesenteric and serosal deposits and subcentimetric lesions were better characterized by DWI-MRI than by CT [55].

In conclusion, peritoneal assessment remains challenging, as both CT and MRI tend to underestimate disease burden compared with surgical findings, but advanced MRI techniques, including DCE-MRI and DWI, show potential to predict tumor invasiveness and metastatic implantation.

Overall, MRI is considered a useful complement to CT, improving restaging accuracy and supporting more appropriate treatment decisions in patients with PDAC.

## Conclusions

The assessment of response to NAT in pancreatic ductal adenocarcinoma remains a complex, still partially unresolved issue, mainly due to the tumor’s intrinsic biological characteristics and its heterogeneous response to treatment.

Conventional morphologic imaging criteria, although widely used, show significant limitations in distinguishing viable tumor from treatment-induced fibrosis and in accurately evaluating vascular and nodal involvement. In this scenario, MRI emerges as a promising complementary modality, enabling evaluation of tumor microstructure through advanced techniques such as DWI, DCE-MRI, and extracellular space quantification.

Despite encouraging preliminary results, these approaches are still limited by technical variability, lack of standardization, and insufficient validation in large prospective cohorts. Therefore, further research is needed to establish reproducible imaging features and to integrate functional imaging into standardized response assessment criteria. A multimodal approach combining morphological, functional, and clinical parameters currently represents the most effective strategy for patient selection and surgical decision-making.

## Abbreviations

ECV: Extracellular volume
NAT: Neoadjuvant therapy
PDAC: Pancreatic ductal adenocarcinoma
USPIO: Ultra-small superparamagnetic iron oxide
